# Analysis of the therapeutic effect of Dimu Ningshen (TCM formula) on attention deficit hyperactivity disorder based on gut microbiota and serum metabolomics

**DOI:** 10.1186/s12906-022-03512-5

**Published:** 2022-01-25

**Authors:** Kairui Tang, Wenzhi Hao, Xiaowei Mo, Yueyue Chen, Xiaofang Guo, Liangliang He, Binghua Wang, Juxian Wang, Qingyu Ma, Lijuan Deng, Jiaxu Chen

**Affiliations:** 1 Guangzhou Key Laboratory of Formula-Pattern of Traditional Chinese Medicine, School of Traditional Chinese Medicine, Jinan University, Guangzhou, China; 2 College of Pharmacy, Jinan University, Guangzhou, China; 3 Xiangnan Traditional Chinese Medicine Osteopathic Hospital, Yantai, China

**Keywords:** Attention deficit hyperactivity disorder, Chinese medicine compound, Gut microbiota, Nontargeted metabolomics

## Abstract

**Background:**

Attention deficit hyperactivity disorder (ADHD) is a neurodevelopmental disorder diagnosed during adolescence and adulthood. Assessment of the long-term risks of the current drugs for ADHD treatment has been insufficient, and little is known concerning the long-term therapeutic effects of psychostimulants. Commercially available traditional Chinese medicine compound oral preparations [e.g., Dimu Ningshen (DMNS)] have been widely used in the clinical treatment of ADHD, but their influence on the interaction between gut microbes and potential metabolomes remains inconclusive.

**Methods:**

We used a series of behavioral experiments to evaluate the behavioral effects of DMNS on adolescent and adult ADHD rats and used 16S rDNA sequencing of gut microbes and nontarget metabolomics to evaluate the potential pathogenesis of ADHD and explore the biological mechanism of DMNS in ADHD treatment.

**Results:**

For the first time, DMNS was shown to reduce the excessive activity of adult and adolescent ADHD rats and improve the attention deficit of adult ADHD rats. DMNS improved the structural composition of the ADHD gut microbiota and reduced the abundance of *Ruminococcaceae_NK4A214_group*, *Ruminococcus_2*, and *Eubacterium_nodatum_group*. Simultaneously, DMNS increased the circulating levels of peripheral monoamine neurotransmitter precursors (e.g., phenylalanine) and reduced the circulating levels of peripheral fatty acid amides (e.g., oleamide). Finally, the changes in the ADHD serum metabolites were strongly correlated with the gut microbiota.

**Conclusion:**

DMNS has a good effect in treating ADHD, and it may exert this effect by regulating the gut microbiota and affecting metabolites in the peripheral circulation.

**Supplementary Information:**

The online version contains supplementary material available at 10.1186/s12906-022-03512-5.

## Background

Attention deficit hyperactivity disorder (ADHD) is a common neurodevelopmental disorder in adolescents and adults that presents with inattention, hyperactivity, and impulsivity and affects more than 190 million people globally [1–3]. With the increasing incidence of ADHD, this neurodevelopmental disorder has already caused significant impairment, has increased annual care costs, and represents a significant economic burden worldwide [4]. Current drug treatments for this disease are limited and have many toxic side effects [5]. Similar to other neurodevelopmental disorders, the cause of ADHD is primarily abnormal neurodevelopment, affecting neurogenesis, synaptogenesis, myelination, and neuronal and glial proliferation and migration [6]. Additionally, norepinephrine (NE), 5-hydroxytryptamine (5-HT), dopamine (DA) and other monoaminergic neurotransmitter dysfunctions play crucial roles in the pathophysiology of ADHD [7].

Extensive evidence indicates that the gut microbiota plays an essential role in the pathophysiology of ADHD [8, 9]. Changes in the gut microbiota have been reported in patients with ADHD, and similar results have been observed in rodents with these conditions [10–12]. Probiotic treatment can improve the clinical symptoms of patients with ADHD [13]. These findings indicate that gut microbes are involved in the development of ADHD and are a potential target for drugs used to manage these conditions [14]. However, the precise role of the gut microbiota in the pathogenesis of ADHD is unknown, and the downstream physiological mechanisms by which gut microbes influence adolescent and adult behavior remain unclear.

Metabolomics is recognized as a powerful approach to characterize the exposome because it can measure thousands of small molecules in biospecimens [15, 16]. This approach has been used to identify mental diseases related to microbial/human metabolism, including Alzheimer’s disease, depression, and anxiety [17, 18]. The bottom-up regulation of the central nervous system by the gut microbiota often depends on its specific metabolites [19]. Accumulating evidence suggests that the gut microbiota and its metabolites play roles in the pathophysiological mechanisms leading to ADHD symptoms [8]. The gut microbiota may interfere with the catecholaminergic neurotransmission system, leading to ADHD by influencing either their metabolic pathways or expression of genes encoding these neurotransmitter transporters [20]. Therefore, an investigation of the changes in gut microbiota and metabolome may aid in further understanding the pathogenesis of ADHD and treatment mechanism of anti-ADHD drugs.

We are seeking to identify another class of drugs that are safe and effective to treat ADHD in addition to neurostimulants, and we are focusing on botanicals. Many herbal drugs or herbal compounds have been investigated for their nootropic and neuroprotective effects on different central nervous system disorders, revealing that changes in the gut microbiota play a key role in the pharmacological effects of drugs [21]**.** Dimu Ningshen (DMNS) is an oral preparation of a traditional Chinese medicine compound that has been on the market and has been used in clinical research to treat children with ADHD [22–24]. However, modern pharmacological studies remain unclear regarding the use of DMNS to treat ADHD. This study aimed to investigate further whether DMNS exerts anti-ADHD effects by modulating the gut microbiota.

## Methods

### Animals and drugs

#### Animals

Specific pathogen-free (SPF) male Sprague–Dawley (SD) rats (3 weeks old) and SPF male spontaneously hypertensive rats (SHRs) (3 weeks old) were obtained from Vital River Laboratory Animal Technology Co., Ltd. (Beijing, China). The study was conducted at the formula-pattern research center of Jinan University (Guangzhou, China) and strictly abided by all applicable institution and government regulations regarding the ethical use of animals. The animals were maintained at a controlled temperature (20 ± 2 °C) and humidity (60 ± 10%) with a 12 h light/12 h dark cycle. After 1 week of adaptive feeding, the rats were randomly divided into four groups. I) SD group (*n* = 6): SD rats were administered a specific amount of pure water; II) SHR group (*n* = 6): SHRs were administered a specific amount of pure water; III) SHR + MH group: SHRs were administered methylphenidate hydrochloride (MH; 1.62 g/kg); IV) SHR + DMNS (n = 6): SHRs were administered DMNS (4.05 mg/kg).

#### Drugs

DMNS (10 mL/bottle; batch number: 180509) was purchased from Yantai Juxian Pharmaceutical Co., Ltd. (YanTai, China). DMNS contains the following 11 Chinese medicines: Di Huang, Gou Qizi, Nv Zhenzi, Shan Zhuyu, Wu Weizi, Shan Yao, Zhi Mu, Xuan Shen, Gan Cao, Mu Li and Long Gu. The specific information is shown in Table S1. The DMNS was checked using the http://www.theplantlist.org database. MH (18 mg/tablet; batch number: J20171049) was purchased from Xi’an Janssen Pharmaceutical Co., Ltd. (XiAn, China).

### Behavioral procedures

This study involved four behavioral experiments: the open field test (OFT), Y maze task, novel object recognition test (NORT) and 5-choice serial reaction time task (5-CSRTT). The specific behavioral operation process is described in the materials and methods section of the supplementary materials. Behavioral results were analyzed using professional behavioral analysis software (EthoVision software analysis system X14; Noldus Information Technology, Wageningen, Netherlands).

### Sample collection and related testing

After completing the last behavioral test, the rats were anesthetized with isoflurane using a small animal breathing anesthesia machine (RWD Life Science, ShenZhen, China). Blood samples were collected from the abdominal aorta for blood biochemical analysis, and stool samples were collected from the cecum in a sterile environment. The stool samples were immediately stored at 20 °C until DNA extraction.

### 16S rDNA sequencing

After extracting genomic DNA from the sample, the V3 + V4 region of 16S rDNA was amplified using barcode-specific primers. The primer sequences were as follows: 341F: CCTACGGGNGGCWGCAG; 806R: GGACTACHVGGGTATCTAAT. The purified amplified product was connected to the sequencing adapter to construct a sequencing library and was sequenced on Illumina (Illumina, Inc., USA). After the raw reads were obtained by sequencing, Usearch software was used to filter the low-quality reads first, the double-ended reads were spliced into tags, and then low-quality tags were filtered out. The resulting data were called clean tags. Next, based on the clean tags, clustering was performed using Usearch software to remove the chimera tags detected during the clustering process and obtain the abundance of OTUs and representative sequences of OTUs. The bioinformatics analysis process of 16S rDNA sequencing is described in the materials and methods section of the Supplementary materials.

### Sample processing in nontarget metabolomics

A 100 μl serum sample was placed in an Eppendorf (EP) tube and resuspended in prechilled 80% methanol and 0.1% formic acid. The samples were then incubated on ice for 5 min and centrifuged at 15000 g for 20 min at 4 °C. The sample was then transferred to a fresh EP tube and centrifuged at 15000 g at 4 °C for 20 min. Finally, the supernatant was injected into a liquid chromatography–tandem mass spectrometry (LC–MS/MS) system for analysis. Subsequent ultrahigh performance liquid chromatography–MS/MS analysis and metabolite matching analysis are described in the materials and methods section of the Supplementary materials.

### Bioassays

The alanine aminotransferase (ALT), total bile acid (TBA), L-lactate dehydrogenase (LDH-L), creatinine (Cre) and triglyceride (TG) levels in serum were detected using an automatic biochemical analyzer (Hitachi, Ltd., Japan).

### DMNS sample processing before UPLC-Q/TOF-MS analysis

One milliliter of DMNS oral solution was added to 3 times the volume of methanol for dilution, the mixture was ultrasonicated for 30 min and centrifuged at 13,000 rpm for 10 min, and 2 μl of the supernatant was subjected to ultra-performance liquid chromatography/quadrupole time-of-flight mass spectrometry (UPLC-Q/TOF-MS) analysis. Subsequent UPLC-Q/TOF-MS analysis of DMNS is described in the materials and methods section of the Supplementary materials.

### Statistical analysis

The data were expressed as means ± SEM and were tested for normality and homogeneity of variance using SPSS 22.0 software (IBM Corp., NY, USA). In multiple group experiments using parametric data, the differences between groups were assessed using one-way analysis of variance to determine overall significance. Because many statistical methods are used in the 16S rDNA sequencing of the gut microbiota, nontarget metabolomics, and the combined analysis of the two, the statistical methods were marked in the annotations below the figure. A *P* value < 0.05 indicated significant differences for all statistical tests. Drawing was performed using GraphPad Prism 7.0 software (GraphPad Software, CA, USA) and Adobe Illustrator CC 2018 software (Adobe Systems Incorporated, San Jose, USA).

## Results

### Preliminary characterization of DMNS and its effect on blood biochemistry in ADHD rats

**Fig. 1:**
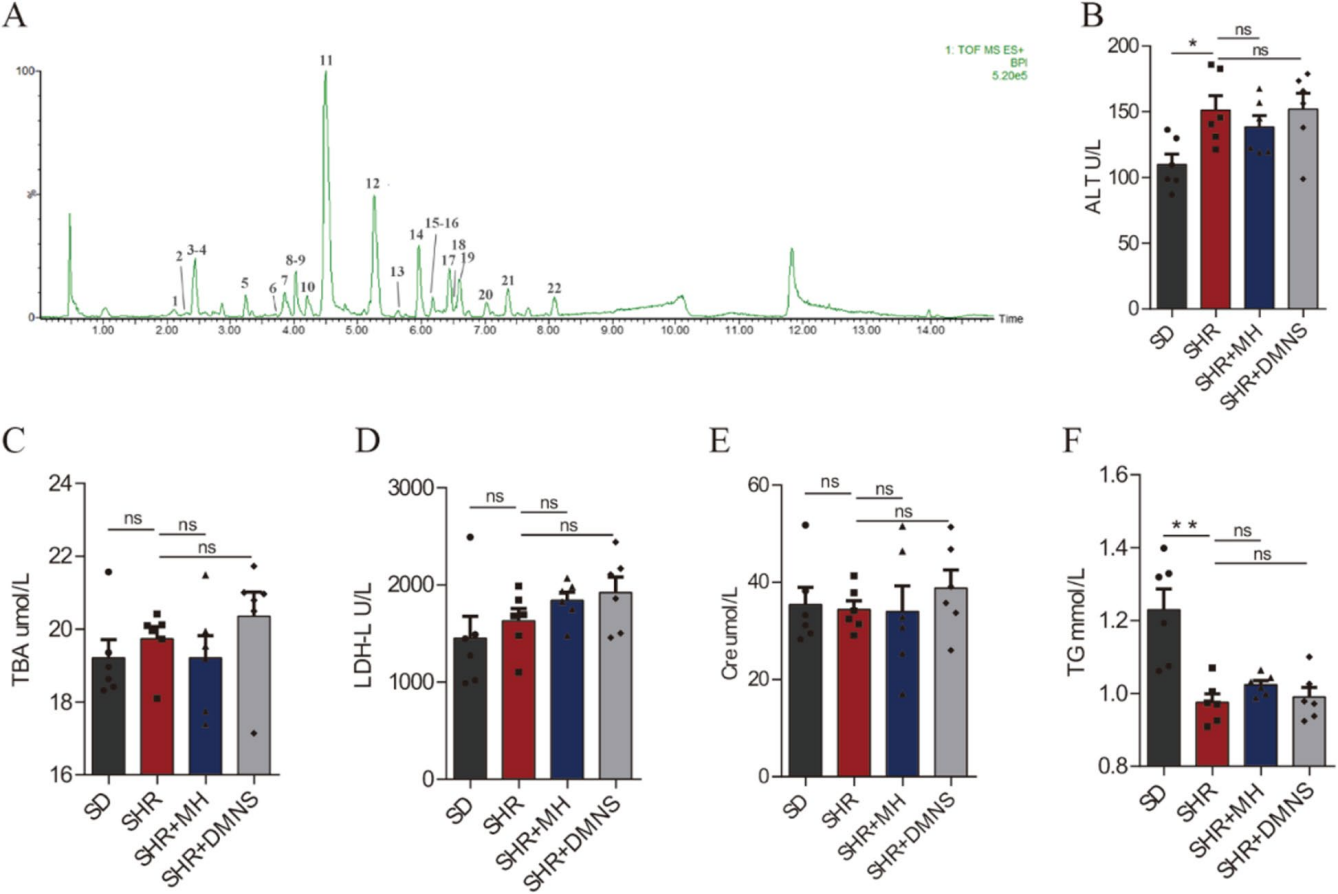
Preliminary characterization of DMNS and the blood biochemical test results of rats in each group. **A** Mass spectrum of the positive ion peak of DMNS. The numbers in the figure indicate the identified compounds, and the specific information is shown in Table S2. **B** ALT level in rat serum. **C** TBA level in rat serum. **D** LDH-L level in rat serum. **E** Cre level in rat serum. **F** TG level in rat serum. One-way analysis of variance (ANOVA) with Tukey’s statistical method. The data were presented as means ± standard error of the mean (SEM) for each group of rats (*n* = 6); *P < 0.05, **P < 0.01

In this study, DMNS was used as a commercial oral solution. To ensure the rigor of the experiment and repeatability in subsequent experiments, we used UPLC-Q/TOF-MS to initially characterize the components of DMNS (Fig. 1A and Fig. S2). Surprisingly, significant differences were found between the SD and SHR groups in ALT (reflecting liver damage) and TGs (reflecting lipid metabolism) (Fig. 1B and F). The level of TGs in the SHR group was significantly lower than that in the SD group (*P* < 0.01), while the level of ALT was higher than that in the SD group (*P* < 0.05). However, no study has shown that this difference was due to the rat breed or onset of ADHD. The circulating levels of TBA, LDH-L, and Cre in the serum of the SHR group were not significantly different from those in the SD group (Fig. 1C, D, and E). After DMNS or MH treatment, the circulating levels of ALT, TBA, LDH-L, Cre, and TGs in the serum did not change significantly compared with those in the SHR group. The above results indicate that DMNS does not damage the liver function, kidney function, or heart function of SHRs.

### DMNS reduces excessive activity in adult and adolescent ADHD rats and improves attention deficits in adulthood

**Fig. 2:**
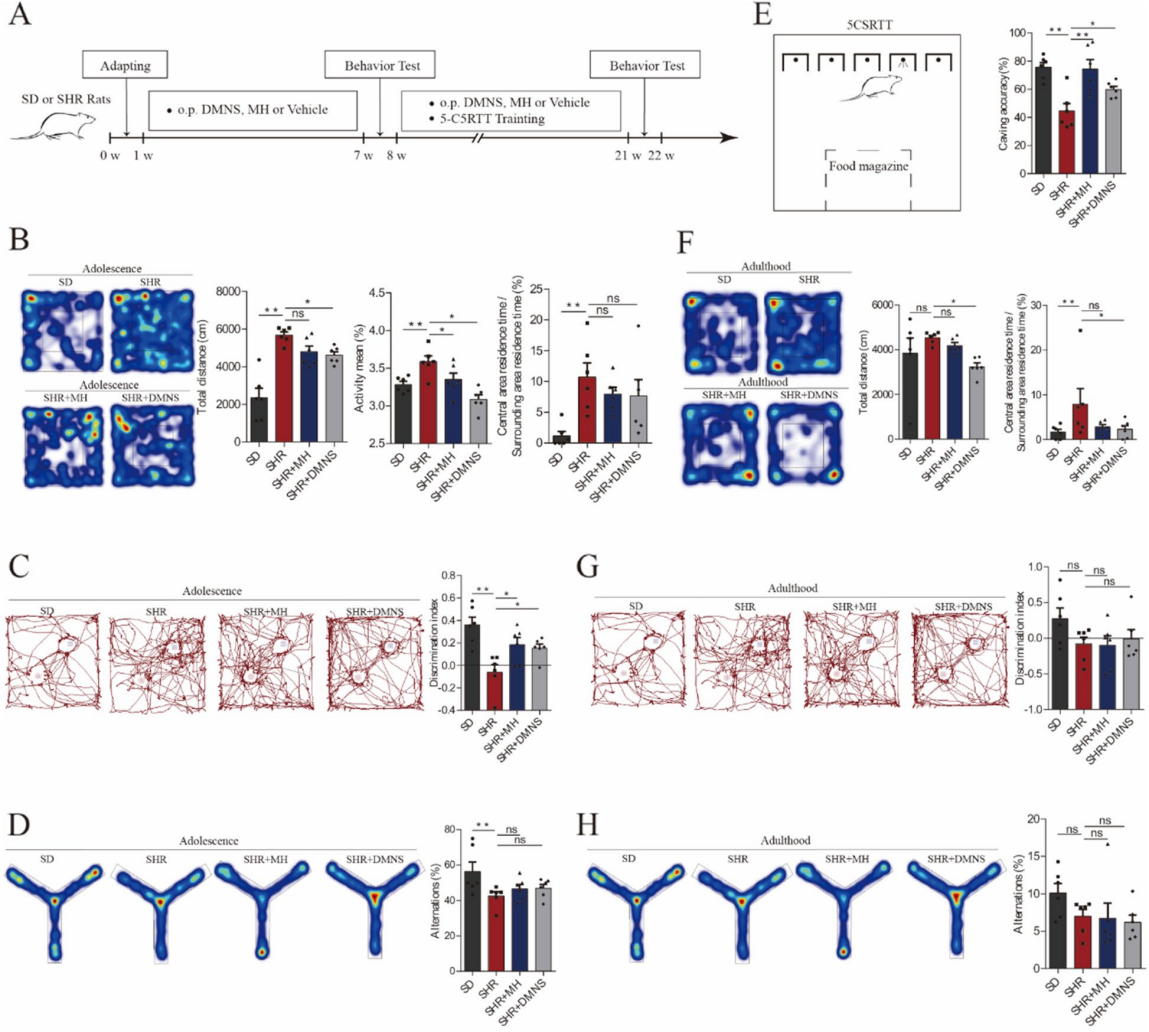
Effect of DMNS on behavior in adult and adolescent ADHD rats. **A** Schematic diagram of the specific experimental process. **B** Open field experiment in adolescent rats. Left: Heatmap of the trajectory of the rat in the open field box; Right: Histogram of data from the open field experiment. From left to right are the total distance, activity, and central area residence time/surrounding area residence time. **C** Novel object recognition experiment in adolescent rats. Left: Trajectory diagram of the rat in the experiment; Right: Distinction index of rats. **D** Y maze experiment in adolescent rats. Left: Heatmap of the trajectory of the rat in the Y maze; Right: Alternations of rats. **E** 5-CSRTT experiment with adult rats. Left: Simple schematic diagram of 5-CSRTT; Right: Caving accuracy of rats. **F** Open field experiment in adult rats. Left: Heatmap of the trajectory of the rat in the open field box; Right: Histogram of data from the open field experiment. From left to right are the total distance and central area residence time/surrounding area residence time. **G** Novel object recognition experiment in adulthood rats. Left: Trajectory diagram of the rat in the experiment; Right: Distinction index of rats. **D** Y maze experiment in adult rats. Left: Heatmap of the trajectory of the rat in the Y maze; Right: Alternations of rats. One-way ANOVA with Fisher’s LSD test was used as the statistical method. The data were presented as means ± SEM for each group of rats (*n* = 6); *P < 0.05, **P < 0.01

Based on the current clinical reports, ADHD is not only a behavioral problem confined to childhood and adolescence. Many scholars have conducted follow-up studies and have demonstrated that among 5–75% of patients, symptoms, particularly social functions, were not alleviated after adolescence or adulthood [25]. To evaluate the behavioral effects of DMNS in ADHD rats in adulthood and adolescence, we conducted an experiment for up to 22 weeks, the specific procedure of which is shown in Fig. 2A.

In the 6th week, the adolescent SHRs showed significant hyperactivity behavioral performance in the open field experiment, and their stay in the central area during the same time was significantly higher than that of the SD rats (*P* < 0.01) (Fig. 2B). After 6 weeks of short-term treatment, DMNS and MH improved the hyperactivity behavior of SHRs in the novel environment to varying degrees, including reducing the total distance traveled and lowering the degree of activity (*P* < 0.05). However, neither drug reduced the residence time of SHRs in the central area. To evaluate the learning and memory abilities of rats quickly and effectively [26], we performed novel object recognition experiments in each group of rats (Fig. 2C). Compared with adolescent SD rats, SHRs in adolescence did not have a significant preference for novel objects (*P* < 0.05), indicating that the learning and memory abilities of SHRs in adolescence decreased. DMNS and MH increased the preference of SHRs in adolescence for novel objects and improved their learning and memory abilities. The Y maze experiment can reflect the spatial recognition ability of rats [27]. The spatial recognition ability of SHRs in adolescence was lower than that of SD rats. Although DMNS and MH improved the spontaneous alternation behavior of SHRs in the Y maze, no significant statistical significance was found (Fig. 2D). The above results indicate that DMNS has a good effect on ADHD rats in adolescence, reducing hyperactivity in the novel environment and improving learning and memory abilities.

In addition to excessive activity, patients with ADHD may have another core symptom, inattention. In this study, we used the 5-CSRTT experiment to evaluate the attention of rats, as shown in Fig. 2E. In the study, the rats received up to 13 weeks of training. Before the formal test, the rats established a stable conditioned reflex between the light source signal and sugar pill. The accuracy of caving in adult SHRs was lower than that in the SD group (*P* < 0.01). The accuracy of caving in the SD group was 75.65%, while that of caving in the SHR group was only 44.56%. SHRs in adulthood exhibited the characteristics of inattention. After long-term administration, both DMNS (*P* < 0.05) and MH (*P* < 0.01) improved the accuracy of burrowing in adult SHRs by 59.79 and 74.41%, respectively, and the improvement ratios were 34.18 and 66.99%, respectively. At the same time, using open field experiments, the total movement distance of SHRs in adulthood was not significantly different from that of SDs, but the residence time of SHRs in the central area was still greater than that of the SD rats (*P* < 0.05) (Fig. 2F). After DMNS treatment, the total movement distance and residence time in the central area of SHRs in adulthood were significantly reduced (*P* < 0.05). In the novel object recognition experiment and Y maze experiment, no significant difference was found in the distinguishing ability or spontaneous alternation rate of SHRs in adulthood compared with that observed in the SD rats (Fig. 2G and H). Additionally, the distinguishing ability and spontaneous alternation rate of adult SHDs treated with DMNS did not increase significantly.

### Effect of DMNS on the gut microbiota structure of ADHD rats

**Fig. 3:**
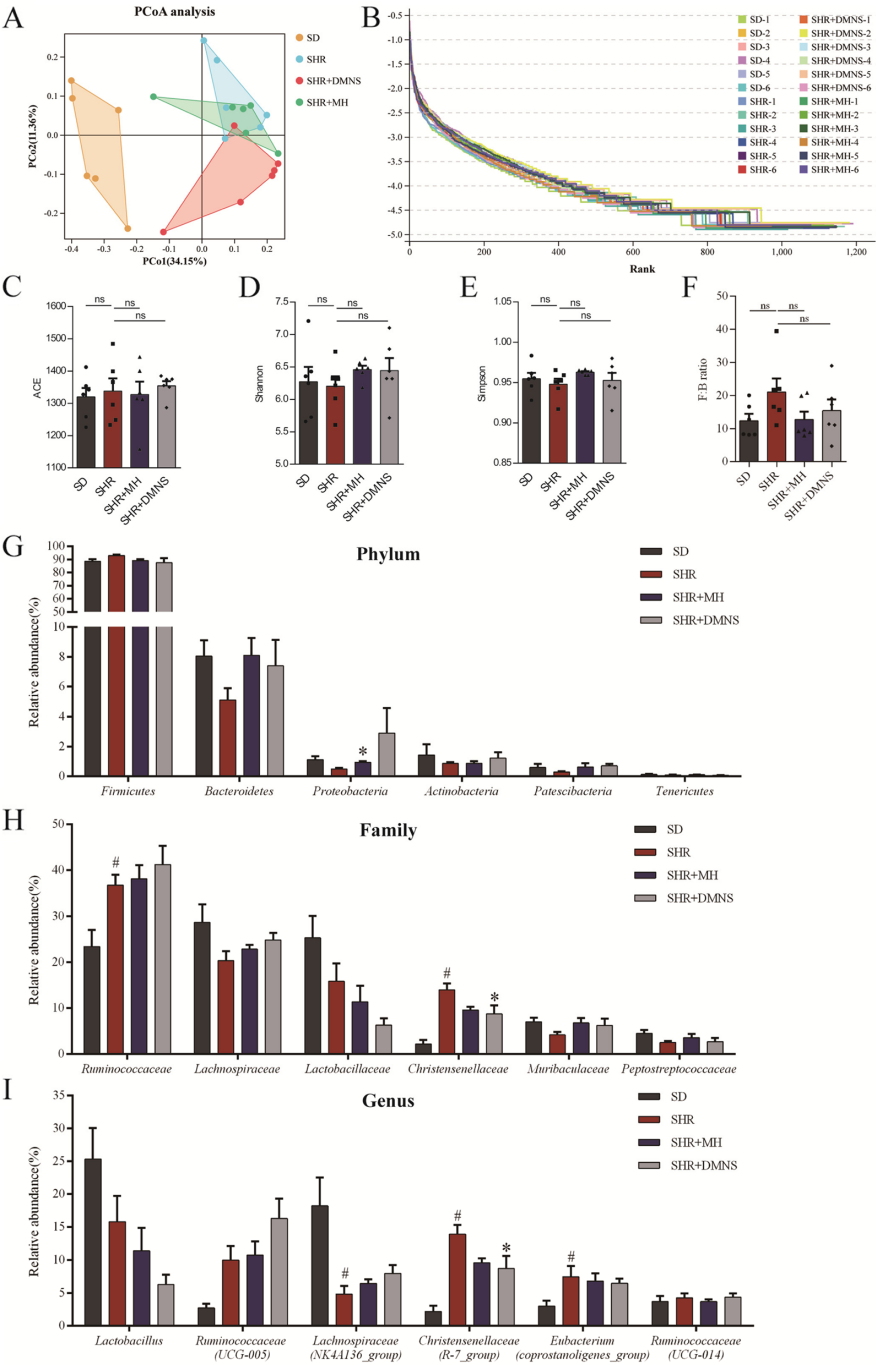
DMNS affected the composition of the gut microbiota of ADHD rats. **A** PCoA of the gut microbiota. The degree of explanation of the overall difference in bacterial colony structure was evaluated by each coordinate axis as a percentage. **B** Rank abundance curve of the gut microbiota. The horizontal axis is the operational taxonomic unit (OTU) rank number (rank) in the corresponding sample, and the vertical axis is the log10 of the relative abundance value of the corresponding OTU. **C**-**E** Histograms of the abundance-based coverage estimator, Shannon, and Simpson indexes. **F** *Firmicutes*/*Bacteroidetes* ratio at the phylum level. **G** Histogram of the top 6 species abundances at the phylum level. **H** Histogram of the top 6 species abundances at the family level. **I** Histogram of the top 6 species abundances at the genus level. One-way ANOVA with Tukey’s test was used as the statistical method. The data are presented as means ± SEM for each group of rats (*n* = 6); ^#^*P* < 0.05 compared with the SD group; *P < 0.05 compared with the SHR group

To explore the possible biological mechanism of the therapeutic effect of DMNS, we performed 16S DNA sequencing on the cecum contents of the rats. Principal coordinates analysis (PCoA) revealed that the structure of the SD gut microbiota is significantly different from that of the other three groups. The SHR and SHR + DMNS groups had fewer overlapping areas, indicating that DMNS affects the structure of the gut microbiota of the SHRs (Fig. 3A). The rank abundance curve in Fig. 3B intuitively displays the richness and uniformity of the microbiota. The span and smoothness of the four groups of curves in the figure are similar, indicating that the richness and uniformity of the microbiotas of these four groups are similar. We used three α diversity indexes— abundance-based coverage estimator, Shannon, and Simpson—to evaluate the richness of the gut microbiota of each group (Fig. 3C, D, and E). Regardless of the index, no significant difference was found between the groups, indicating that the abundances of the gut microbiota in each group are indeed similar.

To clarify the specific influence of DMNS on the structure of the gut microbiota of SHRs, we will analyze the microbiota at the phylum, family, and genus levels. At the phylum level, no significant differences in the *Firmicutes*/*Bacteroidetes* (F/B) ratio were observed between groups (Fig. 3F). The proportion of *Bacteroidetes* in the SHR group was 5.11%, which was lower than that in the SHR + DMNS group (7.39%) and SHR + MH group (8.10%), but there was no significant difference. The proportion of *Patescibacteria* in the SHR group (0.29%) was lower than that in the SHR + DMNS group (0.71%) and SHR + MH group (0.64%, *P* < 0.05), as shown in Fig. 3G. At the family level, the structure of the SHR group was quite different from that of the SD group. The proportions of *Ruminococcaceae* and *Christensenellaceae* in the SHR group (36.70 and 13.94%, respectively) were significantly higher than those in the SD group (23.39 and 2.19%, respectively, *P* < 0.05), while DMNS downregulated the proportion of *Christensenellaceae* in the SHR + DMNS group (8.72%, *P* < 0.05) (Fig. 3H). At the genus level, the proportions of *Christensenellaceae_R-7_group* and *Eubacterium_coprostanoligenes_group* in the SHR group (13.92, 7.44%) were higher than those in the SD group (2.18, 3.01%, *P* < 0.05), while DMNS downregulated the proportion of *Christensenellaceae_R-7_group* in the SHR + DMNS group (8.70%, *P* < 0.05) (Fig. 3I).

### The differential gut microbiota is associated with ADHD regulated by DMNS

**Fig. 4:**
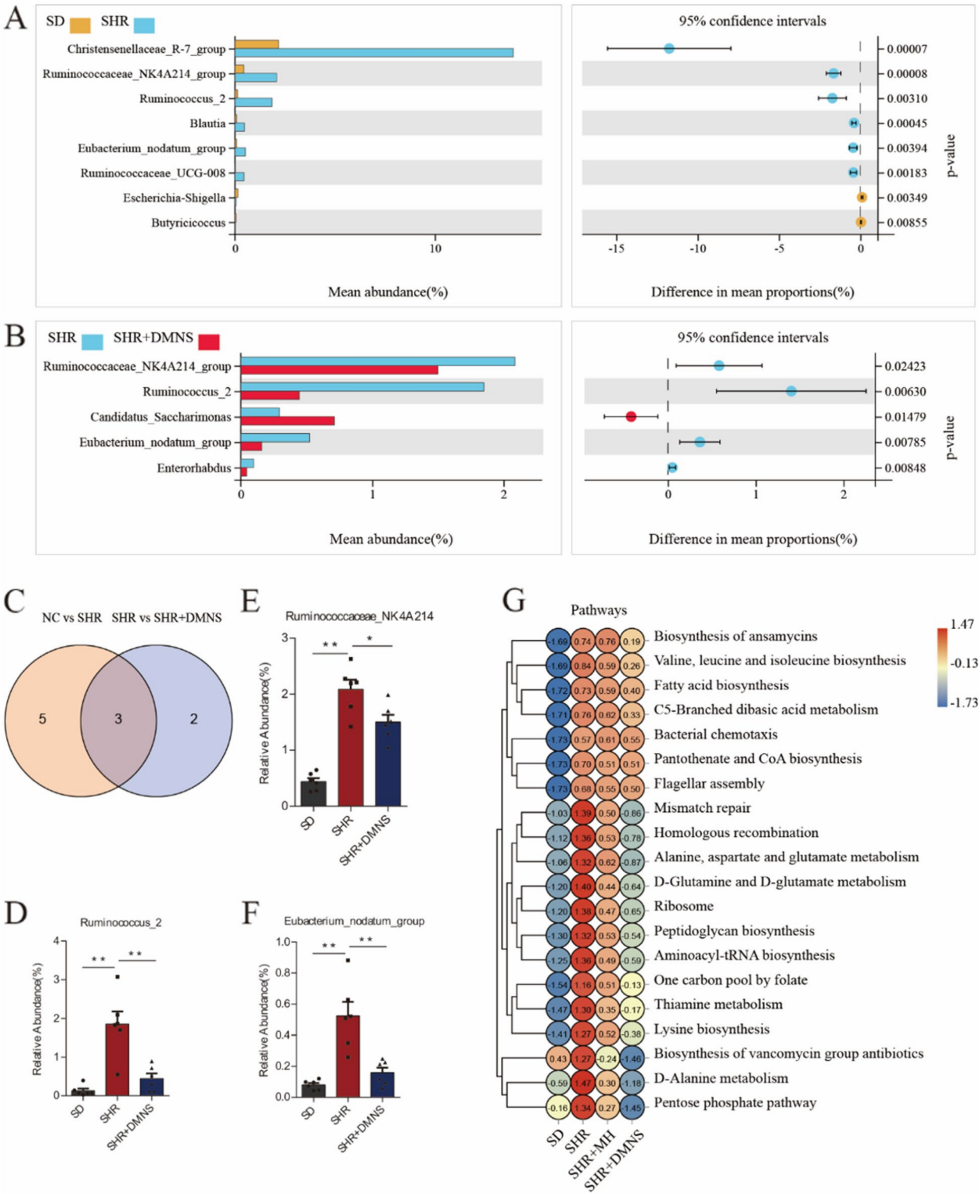
Differences in the gut microbiota between groups. **A** Microbiotas of SD rats vs. SHRs at the genus level (Welch’s t test). **B** Microbiotas of SHRs vs. SHRs+DMNS at the genus level (Welch’s t test). **C** Venn diagram of the different microbiotas after pairwise comparison. **D**-**F** Histograms of *Ruminococcaceae_NK4A214_group*, *Ruminococcus_2* and *Eubacterium_nodatum_group* (Tukey test). **G** Pathways heatmap. The vertical axis indicates the classification of pathways, the horizontal axis indicates the group, and the color indicates the level of abundance. The data are presented as means ± SEM for each group of rats (*n* = 6); *P < 0.05, **P < 0.01

To understand the microbiota differences between each group and clarify the specific regulatory effect of DMNS, we used Welch’s t test to screen the microbiota differences between the SD and SHR groups and between the SHR and SHR + DMNS groups at the genus level (Fig. 4A, B). At the genus level, 8 different microbiotas were identified between the SD and SHR groups and 5 different microbiotas were identified between the SHR and SHR + DMNS groups. The intersection analysis of these different bacteria revealed three key different bacteria—*Ruminococcaceae_NK4A214_group*, *Ruminococcus_2*, and *Eubacterium_nodatum_group* (Fig. 4C-F). These three bacteria had high abundance in the SHR group but low abundance in the SD and SHR + DMNS groups. The analysis of the origin of these three bacteria revealed that they all belong to *Firmicutes* (phylum) and *Clostridiales* (order), strongly implying that the high abundance of *Clostridiales* may be closely related to the onset of ADHD. Additionally, we analyzed the metabolic pathways corresponding to the gut microbiota of each group. The SHR group was mainly enriched in D-alanine, D-glutamine and D-glutamate metabolism and fatty acid biosynthesis, while the SHR + DMNS group showed low enrichment in these pathways (Fig. 4G).

### The differential serum metabolites are related to ADHD regulated by DMNS

**Fig. 5:**
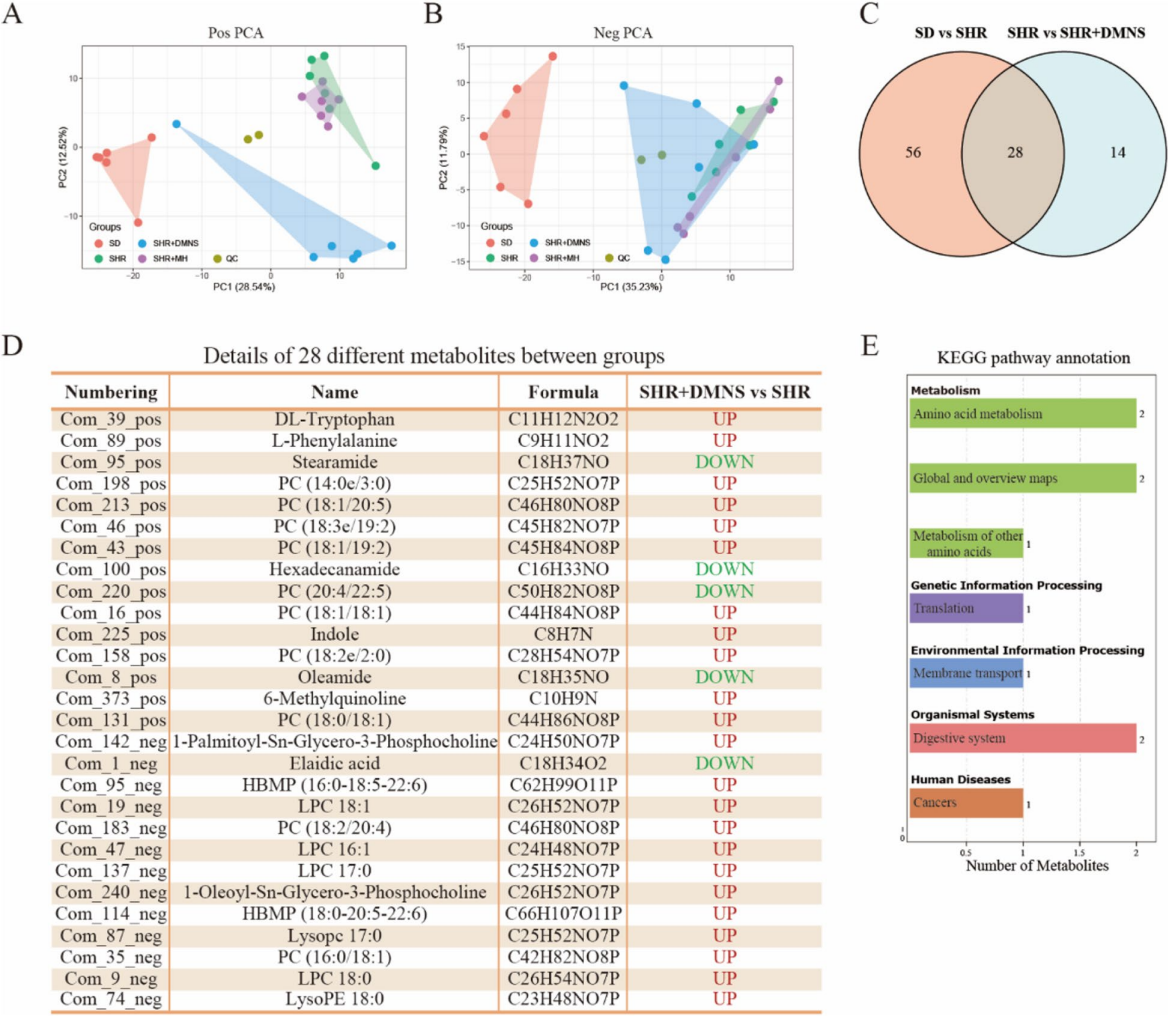
Differences in the serum metabolites between groups. **A**-**B** PCA of metabolites in the positive ion mode and negative ion mode. **C** Venn diagram representing the different metabolites after pairwise comparison. **D** Details of 28 different metabolites between groups. **E** Annotation of the KEGG metabolic pathways of 28 different metabolites between groups

The onset of ADHD may be related to the decrease in the circulating concentrations of metabolites such as tyrosine, phenylalanine, and tryptophan that are involved in neurotransmitter synthesis or branched-chain amino acid metabolism [28]. Therefore, we used nontargeted liquid chromatography–mass spectrometry (LC–MS) metabolomics to evaluate the serum differential metabolites in ADHD rats. PCA was conducted to observe the degree of dispersion between the samples, revealing that in the positive ion mode, the overlapping area of the SHR and SHR + DMNS groups was reduced, and the area of the SHR + DMNS group was closer to that of the SD group (Fig. 5A). However, in the negative ion mode, considerable overlap existed between the SHR and SHR + DMNS groups (Fig. 5B). In general, differences in metabolites were found between the SHR and SHR + DMNS groups. To further explore the differences in metabolites between the groups, we identified 84 metabolites that differed between the SD and SHR groups and 42 metabolites that differed between the SHR and SHR + DMNS groups through relevant statistical analysis methods. After the two were combined, 28 different metabolites were screened out (Fig. 5C, D).

Compared with the SHR group, 23 metabolites in the SHR + DMNS group were significantly upregulated, and 5 metabolites were significantly downregulated. As expected, metabolites involved in the synthesis of neurotransmitters, such as phenylalanine, tryptophan, and indole, exhibited decreased circulating levels in the SHR group, and DMNS increased these metabolites. DMNS can also reduce the content of metabolites such as stearamide, palmitamide, oleamide, and rosin acid. We performed KEGG enrichment analysis of these 28 different metabolites and found that they were mainly enriched in amino acid metabolism (Fig. 5E). This finding was consistent with the metabolic pathways corresponding to the abovementioned differential microbiotas and was also related to amino acid metabolism. This result indicated that in ADHD rats, a strong correlation existed between the different gut microbiotas and different metabolites. Additionally, DMNS may treat ADHD by regulating the metabolites of phenylalanine, tryptophan, indole, and others.

### A strong linear correlation exists between the differential microbiota and differential metabolites

**Fig. 6:**
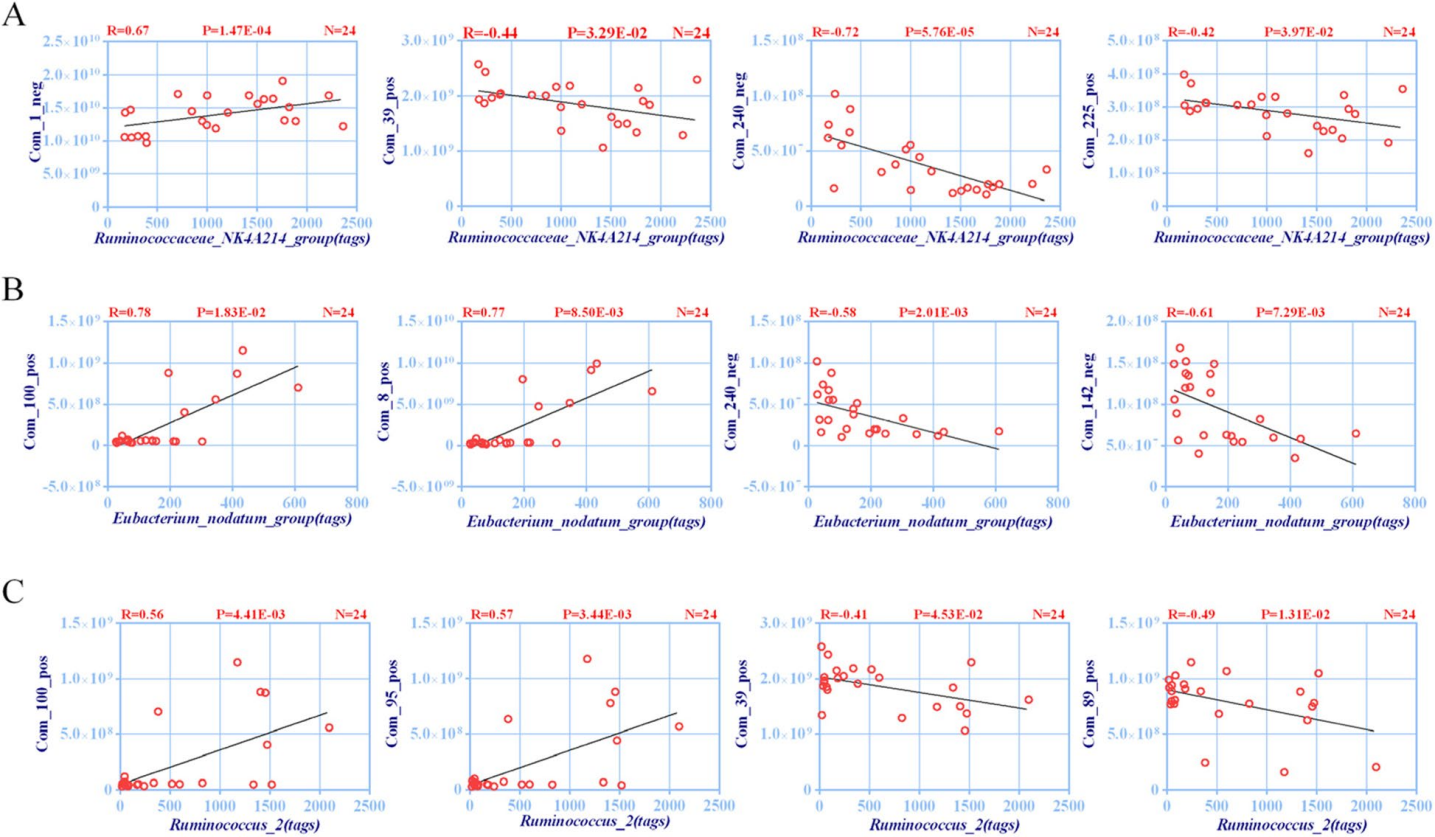
Pearson correlation coefficient (r) and *p* value (p) for the correlation between the differential microbiota and differential metabolites. **A** Pearson correlation coefficients of *Ruminococcaceae_NK4A214_group* and 1-oleoyl-Sn-glycero-3-phosphocholine, DL-tryptophan, indole, and elaidic acid. **B** Pearson correlation coefficients of *Eubacterium_nodatum_group* and oleamide, hexadecanamide, 1-oleoyl-Sn-glycero-3-phosphocholine, and indole. **C** Pearson correlation coefficients of *Ruminococcus_2* and stearamide, hexadecanamide, L-phenylalanine, and DL-tryptophan

To understand the specific correlation between the differential microbiota and differential metabolites, we used the Pearson correlation coefficient to evaluate the relationship between the two (Table S3). *Ruminococcaceae_NK4A214_group* exhibited a significant correlation with 21 different metabolites, among which 1-oleoyl-Sn-glycero-3-phosphocholine, DL-tryptophan, and indole exhibited negative correlations and elaidic acid exhibited a positive correlation (Fig. 6A). *Eubacterium_nodatum_group* exhibited a significant correlation with 24 different metabolites, among which 1-oleoyl-Sn-glycero-3-phosphocholine and indole exhibited a negative correlation and oleamide and hexadecanamide exhibited a positive correlation (Fig. 6B). *Ruminococcus_2* exhibited a significant correlation with 24 different metabolites, among which L-phenylalanine and DL-tryptophan exhibited a negative correlation and stearamide and hexadecanamide exhibited a positive correlation (Fig. 6C).

## Discussion

The overall prevalence of mental illness associated with ADHD in children and adolescents is approximately 40–80%, while 5–75% of adolescents with ADHD still have behavioral symptoms of inattention or impulsivity in adulthood [25, 29]. We explored other safe and effective drugs that exclude neurostimulants represented by methylphenidate that could be used by both adolescents and adults, focusing on natural botanicals. Our study showed for the first time that DMNS reduces ADHD overactivity and improve inattention and is effective for ADHD in adolescents and adults.

At the beginning of the study, we focused on the quality control of DMNS. Thus, we first used UPLC-Q/TOF-MS to initially characterize the composition of DMNS, identifying 22 compounds, which laid the foundation for future experiments. In the present study, we used SHRs (considered an animal model of ADHD) as the experimental group and SD rats as the normal control group [30]. After 4 weeks of age, SHRs usually exhibited ADHD-like symptoms, such as hyperactivity in a novel environment or attention deficit in response to operational conditions. Additionally, the performance of adolescent SHRs in the novel object recognition experiment and Y maze experiment was worse than that of SD rats, indicating that the learning and space exploration ability of adolescent SHRs was significantly impaired. After 6 weeks of DMNS treatment, the excessive activity and learning ability of adolescent SHRs were improved. However, surprisingly, adult SHRs did not seem to show behaviors such as hyperactivity and learning disabilities to a greater extent than SD rats. Anna Kozłowska et al. analyzed the immune, endocrine and nervous systems of adolescent and adult SHRs and found that increased levels of steroid hormones in adult SHRs can alleviate some symptoms of ADHD, possibly explaining why the behavior of adult SHRs in this study differed from that of adolescent SHRs [31]. In the 5-CSRTT experiment, the correct rate of caving in adult SHRs was lower than that in SD rats, indicating that SHRs showed attention deficits in adulthood and that DMNS can improve attention deficits. In summary, the effectiveness of DMNS along with its lack of a significant effect on the blood biochemistry of SHRs indicated that DMNS is a safe and effective drug that can treat ADHD. DMNS can reduce the excessive activity of SHRs in adulthood and adolescence and improve the attention deficit of SHRs in adulthood.

Based on the development of omics methods, the synergistic effect of botanicals and gut microbes significantly affects the health of the host [32]. Studies have shown that supplementation with probiotics can have a positive impact on the progression of neurodevelopmental disorders, including ADHD [33]. Anouk C. Tengeler et al. transplanted the fecal flora of ADHD patients and healthy people to germ-free mice. Germ-free mice showed symptoms similar to those of the donor, and the altered microbial composition may be the driving force for changes in brain structure and function and accompanying changes in animal behavior [34]. As an oral botanical drug, DMNS contains active compounds that interact with the gut microbiota before being absorbed by the gastrointestinal tract [35]. UPLC-Q/TOF-MS analysis revealed 5 timosaponins in DMNS. According to research reports, these saponins have antidepressant and antineuritis pharmacological activities. Guo-Ming Dong et al. found that the gut microbiota has a strong metabolic effect on timosaponin BII, which can be converted into 7 characteristic metabolites [36]. Considering that the phytochemical-microbiota interaction is the key to drug metabolism, we will focus our research on the influence of DMNS on the abundance, diversity and function of the gut microbiota of ADHD rats.

Our study showed that the abundance of the gut microbiota is not significantly related to the pathogenesis of ADHD, but differences exist in the structure of the gut microbiota between ADHD and SD rats. At the family and genus levels, *Christensenellaceae* accounted for a higher proportion in the SHR group than in the SD group. After DMNS treatment, the proportion of *Christensenellaceae* decreased. Considering that *Christensenellaceae* is a universal and heritable strain closely related to obesity, diabetes and other metabolic-related diseases [37], we speculate that *Christensenellaceae* may participate in the pathogenesis of ADHD by influencing metabolic pathways. We screened out different microorganisms using Welch’s t test and found that 3 microorganisms (*Ruminococcaceae_NK4A214_group*, *Ruminococcus_2*, *Eubacterium_nodatum_group*) had high abundance in the SHR group but low abundance in the SD and SHR + DMNS groups. This finding is consistent with that of a Dutch research team investigating ADHD, implying that these three microorganisms may have a special effect on neurodevelopment, a key to brain development and several mental diseases, including ADHD [38].

The brain-gut axis is critical for the gut microbiota to act on the central nervous system of the host. It has a two-way regulatory effect, involving mechanisms including immunity, short-chain fatty acids, neurotransmitter production, the vagus nerve, serotonin, and the endocannabinoid system [39, 40]. One of the pathogeneses of ADHD is related to the insufficient synthesis of monoamine neurotransmitters (dopamine and serotonin) in the central nervous system [41]. Therefore, using neurostimulants to increase the central dopamine content is very effective in improving the symptoms of ADHD. Tryptophan and phenylalanine are involved in the synthesis of monoamine neurotransmitters, including catecholamines and indoleamines, and they are essential amino acids required by the host, indicating they cannot be synthesized by the body itself [42, 43]. The gut microbiota regulates the absorption and metabolism of these monoamine neurotransmitter precursors through the phenylalanine, glutamate, and tryptophan pathways and affect the synthesis and transport of the host’s central nervous system [44]. In this study, we found that the circulating levels of phenylalanine, tryptophan, and indole in ADHD were decreased. Additionally, the circulating levels of phenylalanine, tryptophan, and indole in the serum of ADHD rats were reduced. These metabolites are associated with the gut microbiota and are negatively correlated with *Ruminococcaceae_NK4A214_group*, *Ruminococcus_2*, and *Eubacterium_nodatum_group*. Thus, we speculate that the metabolic activity of these three bacteria will affect the level of amino acids related to monoamine neurotransmitter synthesis in the peripheral circulation and produce ADHD-related behaviors.

Primary fatty acid amides (PFAMs) are a group of lipids that serve as bioactive lipid signaling molecules [45]. Since their initial discovery, fatty acid amides have been considered crucial cell signaling molecules in the brain and central nervous system. Different fatty acid amides bind to different receptors and cause different biological reactions [46, 47]. Many high circulating levels of fatty acid amides (including stearyl amide, palmitoyl amide, and oleamide) were found in the serum of ADHD rats. Endocannabinoids converted from PFAMs interact with other neurotransmitter systems, including the serotonin and dopaminergic systems. Pauline Lafenêtre et al. showed that the brain cannabinoid system comprises endogenous lipid ligands and the synthesis and degradation mechanisms of endocannabinoids play opposite functions in seeking a balance between novelty and behavioral inhibition [48]. In a clinical study, the consumption of cannabis drugs in healthy volunteers weakened response inhibition and increased risk-taking behavior and dysfunction of executive function and motor control inhibition [49]. Therefore, we speculate that excessively high levels of PFAMs weaken the ability of rats to suppress responses and cause them to produce ADHD-like symptoms.

## Conclusion

In summary, the present study demonstrated for the first time that DMNS protected against ADHD in rats. The mechanism underlying the protective effect of DMNS on ADHD may involve, at least in part, the modulation of gut microbiota and its derived metabolites. Hence, our findings may offer information about the application of DMNS to prevent ADHD in rats, but further studies are needed to verify the mechanism of action of DMNS on the “brain-gut” axis of ADHD.

## Supplementary Information

**Additional file 1.**

## Data Availability

All the data generated or analyzed during this study were included in this published article.

## Abbreviations

ADHD: Attention Deficit Hyperactivity Disorder
ALT: Alanine Aminotransferase
Cre: Creatinine
DA: Dopamine
DMNS: Dimu Ningshen
EP: Eppendorf
LC–MS: Liquid Chromatography–Mass Spectrometry
LDH-L: L-Lactate Dehydrogenase
MH: Methylphenidate Hydrochloride
NE: Norepinephrine
NORT: Novel Object Recognition Test
OFT: Open Field Test
PCoA: Principal Coordinates Analysis
PFAMs: Primary Fatty Acid Amides
SD: Sprague Dawley
SHR: Spontaneously Hypertensive Rat
SPF: Specific Pathogen-Free
TBA: Total Bile Acid
TG: Triglyceride
UPLC-Q/TOF-MS: Ultra-Performance Liquid Chromatography/Quadrupole Time-Of-Flight Mass Spectrometry
5-CSRTT: 5-Choice Serial Reaction Time Task
5-HT: 5-Hydroxytryptamine
