# Supplementary material for: Analysis of the therapeutic effect of Dimu Ningshen (TCM formula) on attention deficit hyperactivity disorder based on gut microbiota and serum metabolomics
Source: BMC Complement Med Ther. 2022 Jan 25;22:24. doi: 10.1186/s12906-022-03512-5 (PMC8790860; doi:10.1186/s12906-022-03512-5)
Supplement: Supplementary file 1 — Additional file 1. [file 12906_2022_3512_MOESM1_ESM.docx]

# Content

Supplementary Materials and Methods............................................................................1

Supplementary Figures ....................................................................................................7

Supplementary Tables ......................................................................................................9

References .......................................................................................................................11

# Supplementary Materials and Methods

## Behavioral procedures

Open field test (OFT)

OFT is a commonly used method of measuring exploratory behaviors and general activities of rodents, which can assess the quality of activities and anxiety[[1](#_ENREF_1)]. The OFT was performed on weeks 7 and 21 in our study. Rats were placed in a behavioral operation room for 10 min for adaptation and then moved to the center zone. Camera recording was initiated and timed, and the behavior of the rats was observed for 10 min. Immediately after each experiment, the boxes were cleaned with 75% alcohol. OFT was performed using internationally recognized behavior analysis software (EthoVision software analysis system X14, Noldus Information Technology, Netherlands).

Y Maze Task

The Y-maze is a three-arm horizontal maze (30 cm long and 8 cm wide with walls 15 cm high) in which the three arms are symmetrically separated at 120°. Rats were placed in a behavioral operating room for 10 min for adaptation and then placed at the end of one arm and allowed to shuttle freely between the three arms. Camera recording was initiated and timed, and the behavior of the rat was observed for 10 min. Immediately after each experiment, the boxes were cleaned with 75% alcohol. The Y-maze task was performed using internationally recognized behavior analysis software. Alternation (%) = (Number of alternations/Total arm entries - 2) × 100%[[2](#_ENREF_2)].

Novel Object Recognition Test (NORT)

The whole experiment was divided into three phases: training, recognition training, and testing. Stage 1: The rat was taken out of the cage and placed in the middle of the open field box with its back facing the operator, and the rat was allowed to explore freely for 10 min; Stage 2: Two identical objects (old objects) were placed in the relative object limit of the open field box. Then, 24 h after phase one, the rat was removed from the cage again and placed with the same object as the two previous objects. The rats were allowed to explore freely for 10 min in the center of the open field box from a distance; Stage 3: An object (old object) and a novel object used in the recognition training were placed in the open field box in the object limit phase. Then, 24 h after recognition training, the rat was placed between the new object and the old object, and the rat was allowed to explore freely for 10 min. NORT was performed using internationally recognized behavior analysis software. The duration of time rats spent exploring each object (the duration of time of exploring familiar object, N1; the duration of time exploring the novel object, N2) was recorded. The recognition index (%) = N2/ (N2 + N1) × 100%.

5-Choice Serial Reaction Time Task (5-CSRTT)

The 5-CSRTT involves the presentation of a visual stimulus in one of five apertures [[3](#_ENREF_3)]. Rats were required to attend to these apertures and respond with a nose poke into the aperture in which the stimulus was presented. Each session, lasting 30 min or 100 trials (whichever occurred first), began with the delivery of a sugar pill free reward and the illumination of the reward cue light. Test trials commenced after retrieval of the initial free reward. To initiate each subsequent trial, the rat was required to nose poke into the reward port (both after correct and error trials), after which the reward cue light was extinguished, and the intertrial interval (ITI) began. After the ITI, the response cue light was illuminated in a random aperture on the 5-choice panel. A correct response was recorded if the rat nose poked into the illuminated aperture within 5 s (the limited hold). This triggered the illumination of the reward cue light and the release of a sugar pill reward into the reward port. A response into the wrong aperture (an incorrect response), a response during the ITI (a premature response), or no response within the limited time (an omission) were punished with a 5 s time out.

The experiment in this study was mainly divided into two parts: a training part and a testing part. In each training phase, each rat was trained for 15 min a day, and the rat was required to reach the standard for 2 consecutive days or more before it could enter the next training phase. As the rat established a stable conditioned reflex between the light source signal and the delivery of the sugar pill, formal testing could begin. 5-CSRTT was performed using internationally recognized behavior analysis software. We calculated the following performance measures: accuracy was assessed as the percentage of correct responses (% correct trials/attempted trials).

## Bioinformatics analysis of 16s rDNA sequencing

Reads filtering

Raw reads were further filtered according to the following rules using FASTP (version 0.18.0): (1) Removing reads containing more than 10% of unknown nucleotides (N); (2) Removing reads containing less than 50% of bases with quality (Q-value) > 20.

Reads assembly

Paired end clean reads were merged as raw tags using FLASH (version 1.2.11) with a minimum overlap of 10 bp and mismatch error rates of 2 %.

Raw tag filtering

The Noisy sequences of raw tags were filtered under specific filtering conditions to obtain the high-quality clean tags. The filtering conditions are as follows: (1) Break raw tags from the first low quality base site where the number of bases in the continuous low quality value (the default quality threshold is ≤ 3) reaches the set length (the default length is 3 bp); (2) Then, filter tags whose continuous high-quality base length is less than 75% of the tag length.

Clustering and chimera removal

The clean tags were clustered into operational taxonomic units (OTUs) of ≥ 97 % similarity using UPARSE (versio.88 n 9.2.64) pipeline. All chimeric tags were removed using UCHIME algorithm and finally obtained effective tags for further analysis. The tag sequence with highest abundance was selected as representative sequence within each cluster.

Taxonomy annotation

The representative OTU sequences were classified into organisms by a naive Bayesian model using RDP classifier (version 2.2) based on SILVA database (version 132) and UNITE database (version 8.0), with the confidence threshold value of 0.8.

## UHPLC-MS/MS analyses and Metabolite matching analysis

UHPLC-MS/MS analyses were performed using a Vanquish UHPLC system (ThermoFisher, Germany) coupled with an Orbitrap Q ExactiveTMHF-X mass spectrometer (Thermo Fisher, Germany) in Gene Denovo Co., Ltd. (Guangzhou, China). Samples were injected onto a Hypesil Gold column (100×2.1 mm, 1.9μm) using a 17-min linear gradient at a flow rate of 0.2mL/min. The eluents for the positive polarity mode were eluent A (0.1% FA in Water) and eluent B (Methanol).The eluents for the negative polarity mode were eluent A (5 mM ammonium acetate, pH 9.0) and eluent B (Methanol).The solvent gradient was set as follows: 2% B, 1.5 min; 2-100% B, 12.0 min; 100% B, 14.0 min；100-2% B, 14.1 min；2% B, 17 min. Q ExactiveTM HF-X mass spectrometer was operated in positive/negative polarity mode with spray voltage of 3.2 kV, capillary temperature of 320°C, sheath gas flow rate of 40 arb and aux gas flow rate of 10 arb.

The raw data files generated by UHPLC-MS/MS were processed using the Compound Discoverer 3.1 (CD3.1, Thermo Fisher) to perform peak alignment, peak picking, and quantitation for each metabolite. The main parameters were set as follows: retention time tolerance, 0.2 minutes; actual mass tolerance, 5ppm; signal intensity tolerance, 30%; signal/noise ratio, 3; and minimum intensity, 100000. After that, peak intensities were normalized to the total spectral intensity. The normalized data was used to predict the molecular formula based on additive ions, molecular ion peaks and fragment ions. And then peaks were matched with the mzCloud(https://www.mzcloud.org/)， mz Vaultand MassListdatabase to obtain the accurate qualitative and relative quantitative results. Statistical analyses were performed using the statistical software R (R version R-3.4.3), Python (Python 2.7.6 version) and CentOS (CentOS release 6.6).

## UPLC-Q/TOF-MS analysis of DMNS

A BEH RP C18 column (2.1mm×100 mm, 1.7 μm) was used to achieve chromatographic separation at a column temperature of 40 °C. The mobile phase consists of water (A) and acetonitrile (B), both of which contain 0.1% formic acid (v/v). Gradient elution was performed at a flow rate of 0.4 mL/min, and the elution procedure was as follows: 5% B, 0-0.5 min; 5-80% B, 0.5-10 min; 80-100% B, 10-12 min; 100% B, 12-13 min; 100%-5% B, 13-14 min; 5% B, 14-15 min. The injection volume is set to 2 μL. The UPLC system is connected in series with a quadrupole time-of-flight mass spectrometer (SYNAPT G2 HDMS, Waters, Manchester, U.K.). The mass spectrometer uses an electrospray ion source (ESI), capillary voltage 3 kV (ESI+) or -2.5 kV (ESI-), cone voltage 30 V (ESI+) or 40V (ESI-), secondary cone voltage 4 V, source Temperature 100 °C, desolvent gas temperature 300 °C, backflush gas flow rate 50 L/h, desolvent gas flow rate 800 L/h, argon is collision gas, in MSE mode, argon is used as collision gas for CID. Data collection is in centroid mode.

# Supplementary Figures


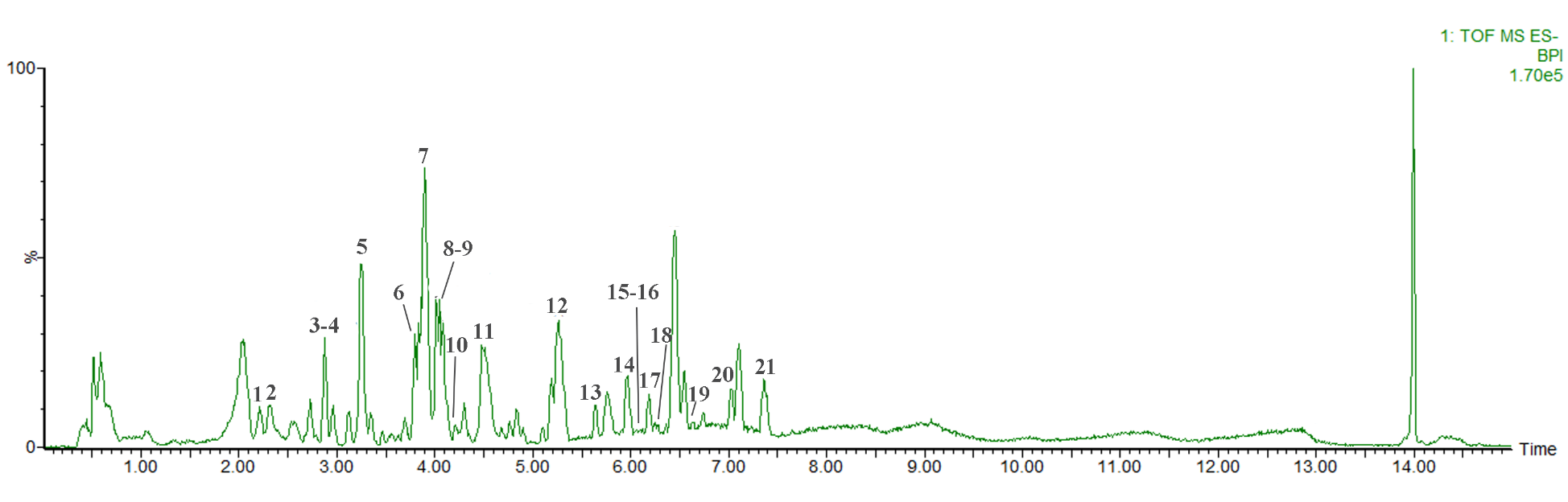


Figure S1. The preliminary characterization of DMNS. The mass spectrum of the negative ion peak of DMNS. The numbers in the figure indicate the compounds that have been identified, and the specific information is shown in Table S1.


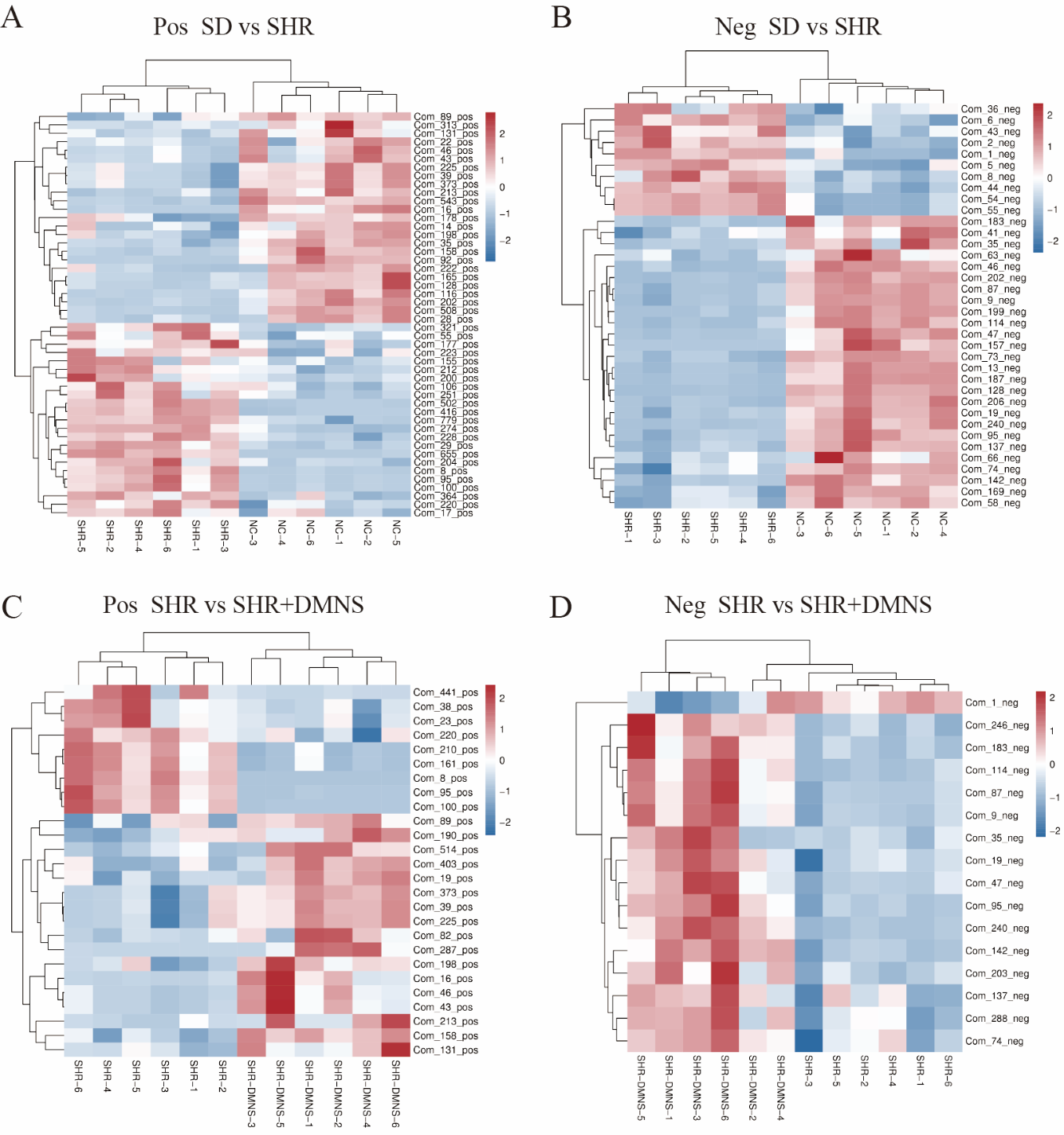


Figure S2. Cluster heat map of differential metabolites. (A) In the positive ion mode, the cluster heat map of the different metabolites between the SD group and the SHR group; (B) In the negative ion mode, the cluster heat map of the different metabolites between the SD group and the SHR group; (C) In the positive ion mode, the cluster heat map of the different metabolites between the SHR group and the SHR+DMNS group; (D) In the negative ion mode, the cluster heat map of the different metabolites between the SHR group and the SHR+DMNS group.

# Supplementary Tables

Table S1. DMNS components.

| **Chinese name** | **Latin name** |
| --- | --- |
| Di Huang | Rehmanniae Radix |
| Gou Qizi | Lycii Fructus |
| Nv Zhenzi | Fructus Ligustri Lucidi |
| Shan Zhuyu | Cornus Officinalis Sieb. Et Zucc. |
| Wu Weizi | Schisandrae Chinensis Fructus |
| Shan Yao | Rhizoma Dioscoreae |
| Zhi Mu | Anemarrhenae Rhizoma |
| Xuan Shen | Figwort Root |
| Gan Cao | licorice |
| Mu Li | Ostreae Concha |
| Long Gu | Os Draconis |

Note: In this study, we used a commercial oral solution. The drug ratio of oral liquid involves the manufacturer's patent information, so it is not mentioned in the manuscript. But we complete the quality control of DMNS through UPLC-Q/TOF-MS technology.

Table S2. Specific information of DMNS-derived compounds that have been marked in the positive and negative ion peaks.

| **Numbering** | **Elemental composition** | **Identification** | **From** |
| --- | --- | --- | --- |
| 1 | C19H1O11 | Isomangiferin | ZM |
| 2 | C45H76O20 | Timosaponin N | ZM |
| 3 | C45H76O20 | Macrostemonoside J | ZM |
| 4 | C45H76O19 | Timosaponin B II | ZM |
| 5 | C45H74O18 | Timosaponin B or C | ZM |
| 6 | C42H62O17 | Macedonoside A | GC |
| 7 | C39H66O14 | Anemarrhenasaponin I | ZM |
| 8 | C42H62O17 | Licoricesaponin G2 | GC |
| 9 | C42H62O17 | Ulasaponin U/N | GC |
| 10 | C42H62O16 | 18a-Glycyrrhizin | GC |
| 11 | C42H62O16 | Glycyrrhizin | GC |
| 12 | C24H32O7 | Schisandrin | WWZ |
| 13 | C39H64O13 | Timosaponin A III | ZM |
| 14 | C39H64O13 | Timosaponin A III Isomer | ZM |
| 15 | C23H26O7 | neokadsuranin | WWZ |
| 16 | C17H26O11 | Morronisid | SZY |
| 17 | C17H24O11 | 8-Epikingiside | NZZ |
| 18 | C17H26O10 | Loganoside | SZY |
| 19 | C16H24O10 | Loganin acid | SZY |
| 20 | C31H42O17 | Nuzenide | NZZ |
| 21 | C29H36O15 | Acteoside | NZZ |
| 22 | C29H36O15 | Isoacteoside | NZZ/DH |

Note. ZM: Zhi Mu; GC: Gan Cao; WWZ: Wu Weizi; SZY: Shan Zhuyu; NZZ: Nv Zhenzi; DH: Di Huang.

Table S3. Pearson correlation coefficients between *Ruminococcaceae_NK4A214_group*, *Eubacterium_nodatum_group*, *Ruminococcus_2* and 28 different metabolites.

| **Differential metabolites** | ***Ruminococcaceae_***  ***NK4A214_group*** | | ***Eubacterium_***  ***nodatum_group*** | | ***Ruminococcus_2*** | |
| --- | --- | --- | --- | --- | --- | --- |
|  | **R** | **P-value** | **R** | **P-value** | **R** | **P-value** |
| Com_1_neg | 6.69E-01 | 3.49E-04 | 5.33E-01 | 3.60E-03 | 4.94E-01 | 1.41E-02 |
| Com_100_pos | 2.69E-01 | 2.03E-01 | 7.89E-01 | 1.83E-02 | 5.60E-01 | 4.41E-03 |
| Com_114_neg | -7.18E-01 | 7.86E-05 | -5.98E-01 | 1.53E-03 | -6.27E-01 | 1.05E-03 |
| Com_131_pos | -4.36E-01 | 3.33E-02 | -4.02E-01 | 8.12E-03 | -5.04E-01 | 1.21E-02 |
| Com_137_neg | -7.60E-01 | 1.63E-05 | -4.78E-01 | 2.31E-03 | -5.06E-01 | 1.16E-02 |
| Com_142_neg | -6.99E-01 | 1.47E-04 | -6.11E-01 | 7.29E-03 | -6.24E-01 | 1.11E-03 |
| Com_158_pos | -7.14E-01 | 8.79E-05 | -5.25E-01 | 8.70E-03 | -4.96E-01 | 1.37E-02 |
| Com_16_pos | -5.34E-01 | 7.18E-03 | -4.29E-01 | 1.01E-02 | -3.95E-01 | 5.63E-02 |
| Com_183_neg | -5.86E-01 | 2.62E-03 | -5.89E-01 | 2.47E-03 | -6.03E-01 | 1.83E-03 |
| Com_19_neg | -7.25E-01 | 6.17E-05 | -5.18E-01 | 2.47E-03 | -5.74E-01 | 3.39E-03 |
| Com_198_pos | -5.28E-01 | 8.07E-03 | -3.76E-01 | 5.18E-02 | -4.95E-01 | 1.40E-02 |
| Com_213_pos | -4.18E-01 | 4.20E-02 | -5.26E-01 | 4.35E-02 | -5.29E-01 | 7.87E-03 |
| Com_220_pos | 3.92E-01 | 5.80E-02 | 5.86E-01 | 1.58E-02 | 4.88E-01 | 1.54E-02 |
| Com_225_pos | -4.22E-01 | 3.97E-02 | -5.14E-01 | 9.59E-03 | -4.01E-01 | 5.24E-02 |
| Com_240_neg | -7.27E-01 | 5.76E-05 | -5.89E-01 | 2.01E-03 | -5.90E-01 | 2.40E-03 |
| Com_35_neg | -5.77E-01 | 3.18E-03 | -4.72E-01 | 1.27E-05 | -4.80E-01 | 1.76E-02 |
| Com_373_pos | -4.26E-01 | 3.81E-02 | -5.27E-01 | 8.32E-03 | -4.14E-01 | 4.41E-02 |
| Com_39_pos | -4.37E-01 | 3.29E-02 | -5.23E-01 | 2.11E-01 | -4.12E-01 | 4.53E-02 |
| Com_43_pos | -2.15E-01 | 3.12E-01 | -2.65E-01 | 1.97E-02 | -2.35E-01 | 2.69E-01 |
| Com_46_pos | -2.32E-01 | 2.75E-01 | -2.88E-01 | 3.65E-02 | -2.51E-01 | 2.36E-01 |
| Com_47_neg | -6.99E-01 | 1.44E-04 | -4.87E-01 | 2.45E-03 | -5.06E-01 | 1.17E-02 |
| Com_74_neg | -6.57E-01 | 4.90E-04 | -4.15E-01 | 4.56E-06 | -6.22E-01 | 1.17E-03 |
| Com_8_pos | 2.71E-01 | 2.00E-01 | 7.66E-01 | 8.50E-03 | 5.82E-01 | 2.82E-03 |
| Com_87_neg | -7.45E-01 | 2.94E-05 | -5.92E-01 | 3.53E-04 | -6.34E-01 | 8.71E-04 |
| Com_89_pos | -2.60E-01 | 2.20E-01 | -6.69E-01 | 1.72E-01 | -4.99E-01 | 1.31E-02 |
| Com_9_neg | -7.57E-01 | 1.87E-05 | -5.89E-01 | 1.14E-05 | -6.38E-01 | 7.92E-04 |
| Com_95_neg | -7.29E-01 | 5.24E-05 | -5.70E-01 | 2.62E-03 | -6.53E-01 | 5.36E-04 |
| Com_95_pos | 2.66E-01 | 2.09E-01 | 7.69E-01 | 6.99E-02 | 5.73E-01 | 3.44E-03 |

References

1. Gould TD, Dao DT, Kovacsics CE: **The open field test**. *Mood and anxiety related phenotypes in mice* 2009:1-20.

2. Miedel CJ, Patton JM, Miedel AN, Miedel ES, Levenson JM: **Assessment of spontaneous alternation, novel object recognition and limb clasping in transgenic mouse models of amyloid-β and tau neuropathology**. *Journal of visualized experiments: JoVE* 2017(123).

3. Asinof SK, Paine TA: **The 5-choice serial reaction time task: a task of attention and impulse control for rodents**. *Journal of visualized experiments: JoVE* 2014(90).
